# P24 *In vitro* activity of cefepime/enmetazobactam, and comparators, against OXA carbapenemase-positive *Acinetobacter baumannii*

**DOI:** 10.1093/jacamr/dlae217.028

**Published:** 2025-01-23

**Authors:** Jon Ward, Nathalie Dunkel, Juan Quevedo, Ian Morrisey, Anne Santerre Henriksen

**Affiliations:** Advanz Pharma UK, London, UK; Advanz Pharma UK, London, UK; Advanz Pharma UK, London, UK; Antimicrobial Focus, Sawbridgeworth, UK; Maxel Consulting, Jyllinge, Denmark

## Abstract

**Background:**

Cefepime/enmetazobactam (FEP/META) is a new β-lactam/β-lactamase inhibitor combination with broad-spectrum activity against MDR Enterobacterales (including OXA-48-producing isolates) which has demonstrated superiority to piperacillin/tazobactam for the treatment of complicated urinary tract infections. This study was conducted to evaluate the activity of cefepime/enmetazobactam against a collection of *Acinetobacter baumannii* isolates positive for characterized OXA carbapenemases.

**Methods:**

The MICs of cefepime, FEP/META (META tested at a fixed concentration of 8 mg/L) and other relevant agents were determined by broth microdilution according to EUCAST guidelines against a panel of 120 OXA-positive *A. baumannii* collected globally (30 isolates each positive for OXA-23, OXA-24, OXA-51 and OXA-58 carbapenemases).

**Results:**

The *A. baumannii* isolates producing class D carbapenemases were 0.8% susceptible to ciprofloxacin or meropenem, and 22.5% susceptible to gentamicin. At the proposed FEP/META breakpoint of 8 mg/L, the susceptibility of OXA-positive *A. baumannii* isolates was 31.7% showing synergy compared with FEP alone. FEP/META had a limited *in vitro* activity against the most common carbapenem resistant *A. baumannii* (OXA-23 or OXA-24 producers), but it showed promising activity against OXA-58 and OXA-51 producers.

**Conclusions:**

Enmetazobactam displayed a capacity to restore cefepime activity against clinical isolates of OXA-51 and OXA-58 carbapenemase producing *A. baumannii*, rendering many of the isolates that were resistant to commercial comparators susceptible to FEP/META.

